# Recycling of Carbon Fiber Reinforced Composite Polymers—Review—Part 1: Volume of Production, Recycling Technologies, Legislative Aspects

**DOI:** 10.3390/polym13020300

**Published:** 2021-01-19

**Authors:** Andrzej K. Bledzki, Holger Seidlitz, Krzysztof Goracy, Magdalena Urbaniak, Janina J. Rösch

**Affiliations:** 1 Faculty of Mechanical Engineering and Mechatronics, West Pomeranian University of Technology in Szczecin, 70-310 Szczecin, Poland; magdalena.urbaniak@zut.edu.pl; 2 Chair of Polymer-Based Lightweight Design, Brandenburg University of Technology Cottbus—Senftenberg, 03046 Cottbus, Germany; holger.seidlitz@b-tu.de (H.S.); Janina.JR.Roesch@bmw.de (J.J.R.); 3 Polymeric Materials and Composites PYCO, Fraunhofer IAP, 14513 Teltow, Germany; 4 Faculty of Chemical Technology and Engineering, West Pomeranian University of Technology in Szczecin, 70-322 Szczecin, Poland; krzysztof.goracy@zut.edu.pl; 5 Digitalization Production, BMW Group, 80788 Munich, Germany

**Keywords:** carbon fibers, polymer composites, recycling, recycled carbon fibers

## Abstract

The paper presents the current volume of international production and global markets of carbon fiber reinforced polymer composites, also regarding the potential development trends. Examples were provided on how to effectively recycle carbon fiber reinforced polymer composites. Legally binding legislation in the EU on polymer composite recycling was given.

## 1. Introduction

When we published our first investigative papers regarding the recycling of GFRPs (Glass Fiber Reinforced Polymers) in the 1990s [1,2,3,4,5,6], we did not think that after almost 30 years CFRP (Carbon Fiber Reinforced Polymers) recycling would be facing the same problems. There are still no system solutions for either GFRPs or CFRPs. Most technical and media reports inform only about completed individual projects. The sources of waste as well as their volumes were known then and are known now. There are known recycling methods and possibilities of material recovery. However, what has been lacking is acceptance for recycled raw materials, trust in their properties, and reliabilities to deliver their continuous supply. Recycled materials still bear the stigma of poor quality. There are no legal standards or regulations that would regulate what recycled materials can be used in major industrial sectors as there is on standard basis use composite materials and are therefore natural, mass users of such products. If there is no market, recycling is not profitable. What follows on from that are landfill sites of used products/composite products stacked globally in wasteland or along shores, as was pointed out in our earlier papers [7,8], i.e., millions of used composite products waiting for… 

The automotive industry is afraid of some technological issues (production time) and it faces the lack of available recycled carbon fibers for mass applications, e.g., for low waste composite production if modern technologies like tape placement will be used. The estimated time necessary for materials tests and certificates in the automotive industry is seven to nine years [9].

The growing demand for carbon fibers is limited mainly by their price. The demand for carbon fibers is estimated to exceed production output in 2022 by 24,000 t [10]. There are good reasons, therefore, for being interested in the recycling of carbon fibers. Some analysts think that recycled fibers can pose an attractive alternative to brand new fibers. Carbon fiber (CF) production is an energy-consuming process, not without a burden to the environment, it is worth mentioning that the most often used precursor for CF are PAN (polyacrylonitrile) fibers and they are spun with very hazardous chemicals. Production of 1 kg of carbon fibers takes 195–595 MJ/kg which is approximately 10 times more than the production of glass fibers [11]. Recycling carbon fibers with chemical methods take only 38 MJ/kg [11].

Almost 90% of CF are produced from PAN precursor, other precursors are lignin, polyethylene, pitch. Still, they give fibers with modest mechanical properties. Efforts in lowering the cost of carbon fiber manufacturing are already underway mainly to improve energy consumption at different levels of heat treatment. Among others, the research collaboration of Saudi Aramco Technologies Company (AramcoTech, Saudi Arabia) and Aachen University (Germany) has aimed for the last two years at lowering the cost of manufacturing standard modulus carbon fibers by using different petroleum-based precursors. In addition, they will explore ways to overcome technical challenges in polyethylene (PE)-based technology, as well as conduct a techno-economic viability study to outline plans to build the first semi-industrial line in the world. It is targeting a very higher reduction in the carbon fiber manufacturing cost, which could result in accelerating the adoption of carbon fiber-reinforced plastics (CFRP) beyond high-end sectors (e.g., automotive, wind energy, and pipeline) [12].

It was reported on 20 August 2020, that the University of Kentucky (Center for Applied Energy Research, U.S.) and the U.S. Department of Energy’s (DOE) Oak Ridge National Laboratory (ORNL U.S.) are teaming up on a USD 10 million projects to transform coal into high-value carbon fibers and composites. The project, titled “C4WARD: Coal Conversion for Carbon Fibers and Composites” seeks to develop the fundamental and translational science and engineering necessary to create energy-efficient and cost-effective processes for manufacturing carbon fibers with tunable properties. It will address challenges associated with coal processing, variability in coal feedstocks, and carbon fiber manufacturing scale-up from laboratory to semi-production scale [13].

The industry that provides rCFs is relatively newly utilized and has to solve a number of problems. Fiber recycling technologies have been known for years. However, it is also clear that recycled fibers will never completely replace brand new fibers. This is particularly the case when high strength and stiffness are required for elements working under long-term dynamic load.

## 2. Historical Outline

**Figure 1:**
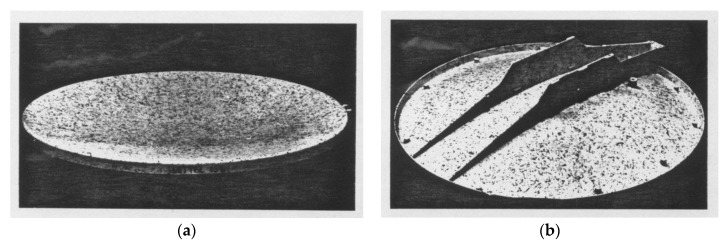
Satellite dish with 15 wt % of Sheet Molding Compound (SMC): (**a**) front view; (**b**) reverse view.

The first research on the recycling of duroplast composites was conducted in Europe in the late 1980s. There was a wave of published papers, diplomas, and dissertations in the early 1990s. Institut für Werkstofftechnik at the University of Kassel headed by Prof. Błędzki was a pioneer. In 1991, the institute produced a diploma on “Duroplast elements using regenerated SMC” [1] that was awarded by the regional Chamber of Industry and Commerce (SMC—Sheet Molding Compound, ready to press polyester composite, delivered as a sheet). The developed recipe for SMC involved the use of regenerated materials (such was the term used then in Germany) and it was implemented for mass-production of a satellite dish (Figure 1).

A few doctoral dissertations concerning the recycling of composites were presented from 1995–1997. At the University of Erlangen on SMC and BMC recycling (BMC—Bulk Moulding Compound, ready to process polyester composite, delivered as a dough-like molding mass). In this work, new formulations of SMC and BMC with mechanically recycled glass fiber reinforced composites were developed and investigated. Moreover, recycled carbon fibers were investigated as a valuable addition for thermoplast composites [14]. At the University of Halle recycling of flame retardant duroplasts used in electronics was investigated. This work presents the mechanical recycling of pressed compounds with a high content of quartz fillers. The possibilities of shredding and obtaining similar materials from the recyclates were presented [15]. A paper published at the Swiss Federal Institute of Technology in Zurich discussed the recycling of composites with a high content of reinforcing fibers, especially unhardened prepreg and thermoplastic composites. As a result, plates with recycled materials were manufactured by pressing [16] and presented at Institut für Werkstofftechnik at the University of Kassel in a dissertation that introduced the possibilities of optimization of SMC recyclate content in composite materials [2]. The results of this work were also described in [3,4,5].

Institut für Werkstofftechnik at the University of Kassel conducted a number of projects in collaboration with Polymer Institute of the then Szczecin University of Technology and Riga Technical University, which brought some published papers [3,4,5]. In [3,5] recyclates were obtained from SMC in the ERCOM process and circuit board recyclates were used to produce SMC with a modified recipe and plates in the RTM process. The maximum possible filling of the composite with recyclate and the basic mechanical properties of the materials obtained were tested. It has been proven that it is possible to add up to 30 weight % of recyclate, following proper modification of the recipe. In paper [4] experimental and theoretical modeling of mechanical properties of materials with recycled particles were presented. An approach based on the planning of experiments was used. This method is based on simple models of material properties as a function of design variables. For materials with recyclate, the weight parts and volume ratios of recyclate fractions with different diameters were design variables. It was shown that for maximum content of recyclate in composite recycled particles with at least three different diameters should be used.

**Figure 2:**
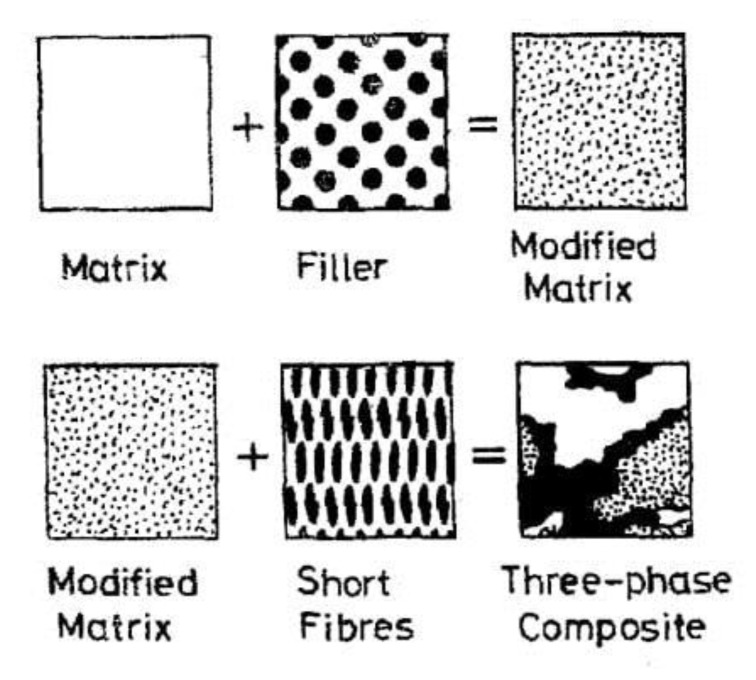
The three-phase model of composite with recycled particles [4].

Additionally, using data of physical experiments and theoretical calculations, a simple mathematical model for composite stiffness was determined [4]. It was assumed that in a composite with recyclate particles there are; phase one—glass fibers of different length (new one and recycled glass), phase two—resin which contains partly recycled particles of resin, and partly new resin added in processing, phase three—filler, only at recyclate particles. This model is presented in Figure 2.

The second step was to calculate the properties of the filler modified matrix. Stiffness E_m_ of the modified matrix was calculated with the formula:

E_m_ = E_r_ {1 + c’_p/_[m/(m − 1) − (c’_p_)^1/3^]} (1)

where: E_m_—modulus of modified matrix; E_r_—modulus of resin; c’_p_—apparent volume fraction of filler; m = E_p_/E_r_ (E_p_ − filler modulus).

This allowed us to use models developed for two-phase composites. However, there was no information about volume fractions of fibers of different length/diameter ratios obtained in the recycling process. Only the weight fractions of new glass fibers and glass fibers from recycled particles were known. To estimate this complex distribution of particles the Hirsch model was used to calculate modulus of composites:

E = α E_V_ + (1 − α) E_R_ (2)

Here, E_V_ modulus is calculated using Voigts’s formula for two-phase composites:

E_V_ = c_f_ E_f_ + (1 − c_f_) E_m_ (3)

and E_R_ modulus is calculated using the Reuss formula for two-phases composites:

E_R_ = E_f_ E_m/_[(1 − c_f_) E_f_ + c_f_ E_m_] (4)

where: E_f_—fiber modulus; c_f_—volume fraction of fibers; α—empirical parameter [4].

Table 1 shows example results of experimental and theoretical investigations for composites with recycled particles [4].

**Figure 3:**
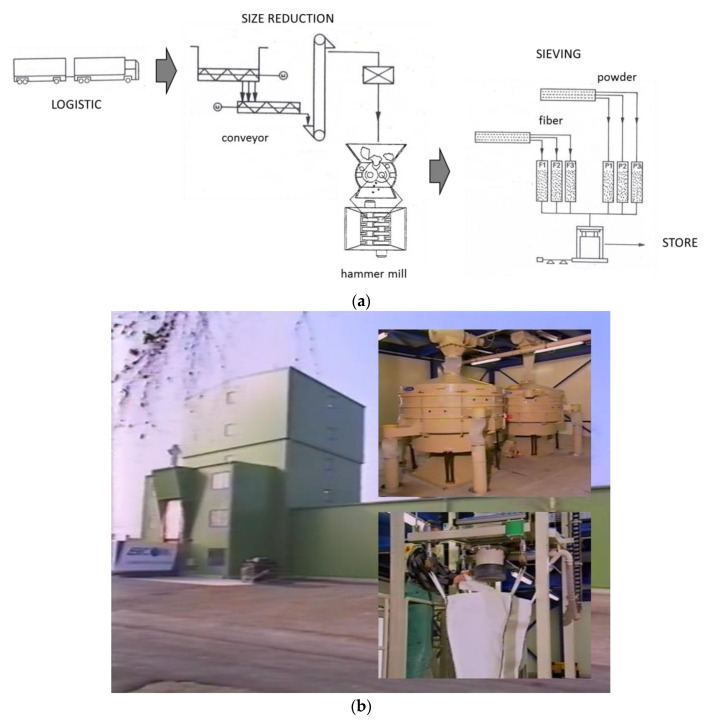
ERCOM installation for SMC/BMC (Bulk Molding Compound) recyclates: (**a**) scheme; (**b**) general view as well as sieving and packing hubs.

Those scientific efforts were undoubtedly triggered by lawmakers and industrial players. Famously, ERCOM was involved in SMC mass processing and shredding. The company was a joint venture of SMC processing players, including BASF, Vetrotex, Menzolit, and Owens-Corning. ERCOM had facilities to produce SMC shredded fractions at 1.5 t/h (approximately 6000 t/year). ERCOM produced recycled particles from used SMC in six fractions; three powder and three fibrous. The size reduction was carried out in several stages; shredding in hammer mill, separation in powder and fiber fractions in two pneumatic cyclones, sieving of each fraction into three more fractions with defined maximum diameters (Figure 3). Unfortunately, the maximum diameter of every fraction was the only known property. The exact properties of fibers and polymer matrix after shredding were not investigated. These properties had to be inferred on the basis of the properties of the shredded materials [2,5].

Similar companies were later set up in Europe: Mecelec Recyclage (France), Valcor, Lonza (Italy), Milijotek (Norway), in the US: R.J. Marshall Company, Premix, Phoenix Fiberglass and in Asia: JRPS (Japan) [17,18,19,20,21].

## 3. Production of Polymer Composites Worldwide

In 2018, the global production of polymer composites reached 11.4 Mt [22]. The largest market (in terms of composite volume) was Asia—5.2 Mt (46%), including China—3.1 Mt (27%), followed by North America—3.0 Mt (26%) and Europe—2.4 Mt (27%). The global market of composites between 1960 and 2010 grew by approximately 8% annually. After 2010, the increase stabilized at roughly 4% per year. The most commonly used materials are duroplast and thermoplast PMCs (polymer matrix composites), which in 2018 accounted for approximately 61 and 39 wt. %. The increase of thermoplast matrix use is particularly noteworthy—from 2% in 1980 up to the present level [22].

Glass fibers are the most commonly used reinforcing material (approximately 87.7%), followed by natural (11%) and carbon fibers (1.2%). Carbon, glass, and natural fibers generated 23%, 68%, and 8% of profit in the sector in 2018, respectively [22].

From 1960, with the global production of composites of 0.2 Mt annually, to 2018, a total of approximately 288 Mt of polymer composites were produced [22].

Despite changing trends in technology and industry, the use of composite materials in various industrial sectors has been at a constant level. Transport and civil engineering consume 36% and 35%, with electrical engineering, electronics (14%), and sport (14%) to follow behind [22].

The volume of polymer composite production in Europe is illustrated in Table 2. Compared to other analyses of the market [6], it is worth making a note that no drops have been recorded in Europe. There are substantial differences between some local markets. For example, Germany is strongly focused on the automotive and electronics industries while the Nordic market is mainly oil and gas.

## 4. CFRP (Carbon Fiber Reinforced Polymer) Market

In 2017, the global demand for carbon fiber was 70,500 t, which was an approximately 11% increase compared to the previous year. The estimated demand for carbon fiber in 2018 was 78,500 t (Table 3) [7,22]. However, profits generated by production could be a better index than production output alone. Since 2013, profits generated from carbon fiber production have been growing by approximately 11% per year and in 2018 amounted to roughly 2.88 billion USD. The world’s biggest producers of carbon fiber are Toray (31% of global output), SGL Carbon, Mitsubishi Chemical Carbon Fiber and Composites, Teijin, and Hexel. 10 companies produce over 87% of global output. Nearly all production of CF is used for polymer composites (approximately 83%). Other matrices for CF composites include ceramic (10%), metal (1%) and other matrix types (approximately 6%) [22]. Within the CFRP-segment thermoset matrix systems play the most important part with an overall turnover of 69%. The share of thermoplastic matrices turnover is 28.8% today [22]. The main thermosetting matrices are polyesters and epoxies. Formaldehyde resins also have a small share. From a recycling point of view, it is of the greatest importance to address the problem of recycling composites with polyester resins.

Since 2010, the global production of CFRPs has been growing by more than 12% annually and in 2018 it was 128 kt (Table 3) [22]. Compared to GFRPs, it does not seem much. However, in 2018, carbon composites generated almost USD 16.5 billion in profits [22]. The majority of carbon composites are used in North America (including Mexico)—37% and in Europe—34%, where many companies specializing in final assembly are based. The consumption of carbon composites (all matrices) in different technological sectors as well as profits are collected in Table 4.

Profits generated from aviation and aeronautics have been brought about by stringent quality standards and costs of certification and qualification of materials and parts. Commercial aviation is the largest customer of carbon composites. New regulations mean that older plane models will have to be replaced and the changes will have to be introduced in currently produced models of airplanes and helicopters.

The automotive industry is the second largest segment—regarding turnover (USD 4.17 bn) as well as the required quantity (37.13 kt). To benefit from the anisotropic fiber structure, new part designs and adapted production technologies are required. The combination of fiber placement technology and HD-RTM reaches the needed readiness level and can be used as a continuous process for producing large series. A further approach is SMC technology with a focus on highly integrative structures [22].

Further developments of industrial application of thermoplastic components have been made within the project MAI Multiskelett with Technische Universität München, P+Z Engineering GmbH, and BMW AG. BMW will replace the steel component between the roof frame and windshield with a thermoplastic fiber-reinforced component. The fiber strands pre-impregnated with thermoplastic material are used with various dimensions by SGL Carbon using its own 50 k carbon fiber. They will be manufactured with pultrusion technology. In a second step, the profiles are then further processed by injection molding into a skeletal plastic component [23,24].

Sport and recreation are sectors that provide a significant part of the profits. Carbon fibers have a well-established position in the sectors right from popular (golf, hockey, cycling, tennis, winter sports) to professional sport and individual solutions (e.g., yachts). Given substantial amounts of carbon fibers and composites in circulation, producers make sure that parts are made in line with recycling rules so that they can be used again. However, current methods of producing parts with carbon fiber composites tend to generate large amounts of waste, which only to a very small degree can be recycled [25,26].

Used parts will soon join this stream of generated waste. Since the question of how to recycle carbon fibers and their composites has not been finally solved, more and more used elements tend to be disposed of in a landfill. If this trend continues, the required amounts of recycled materials might not be met. Depending on the used technology, the amount of carbon fiber waste can reach 30–40%. This currently translates into approximately 24 kt globally and the prediction for 2021 is 32 kt. Less than 1 kt is now recycled [25,26,27]. About 2/3 of fiber waste is generated at the production stage and 1/3 comes from used parts [28].

Unfortunately, not all classical methods of recycling, including material, chemical, and thermal recycling, can be used for carbon fibers and carbon composite products. The simplest solution, i.e., burning of carbon fibers with energy recovery in standard waste incinerators can damage them [29,30]. Practically, carbon fiber recovery is done using pyrolysis and solvolysis.

## 5. Carbon Fiber Recovery Technologies

A systemic solution was developed and implemented by ELG Carbon Fibre Ltd (UK). Specialists from the company claim that recycled carbon fibers retain 90% of their original properties while being 40% cheaper than new fibers [26,27]. What they do therefore is to provide the same properties at a lower price. If all fibers are replaced by rCF in an element its weight can be 5% heavier. In thermoplastic matrices, the properties of recycled fibers are practically the same as those of new materials, without any weight change.

ELG developed a pyrolysis method conducted in a furnace that can process up to 5 tons of waste a day. To ensure continuity of fiber properties, the company checks fiber quality at the inlet and outlet points, and then it classifies fibers in terms of waste type and mechanical properties. Recycled fibers are fluffy and of different lengths. The company divides recycled material into ground fiber suitable for molding compounds and coating (80–100 µm), cut fiber good for thermoplast reinforcement (3–100 mm), and fiber suitable for products with the basis weight of 100–500 g/m^2^ (60–90 mm). Mats are up to 2.7 m wide and can be used to produce prepreg, SMC, and pressured products and semi-products manufactured using different techniques. ELG also produces hybrid mats, where carbon fibers are mixed with thermoplastic fibers, which are used for fast pressing.

ELG claims that their products are not meant to replace new carbon fibers. Instead, they say that they provide a new product that requires new processes and different designs. The firm helps its customers by training them. Thus, it wants to create a market for the new material [26]. Boeing and Mitsubishi are the most important customers of ELG. In 2018 Boeing signed a contract to provide ELG hardened and unhardened materials to be processed, which solved the question of supply continuity. Mitsubishi bought ELG shares and is set to use its own marketing network to promote ELG products [31].

The company claims that rCF can compensate for CF production, which is still too low compared to global demand. A new installation for rCF production can be started within 9 to 12 months. That is twice as fast as the production of new fibers, with a lower total cost. This enables a quick reaction to market demands [31]. Production of rCF generates much fewer greenhouse gases (CO_2_) than the production of new fibers [31]. Unfortunately, all mats, including rCF mats, produce composites where the fiber component is at the level of 40%. High pressure during the manufacturing process is required to obtain higher fiber content [31].

Another problem is how to prepare fiber surface to improve wettability, matrix adhesion, its preparing, and handling. Matters of importance are also limit fatigue damage and environmental resistance sustainability of products. It takes a long time to research these properties and many projects are underway to identify these features for all ELG products [31].

A recent and ambitious project of ELG is to establish a closed-loop recycling process of end-of-life (EoL) aircraft carbon composite material stream, maximizing value via a joint approach in further developing the recycling process and new product development using carbon fiber [32].

Institut für Textiltechnik from Augsburg (Germany) built-in 2016 a pilot installation to produce nonwoven fabrics from recycled polymer composites. The line can use injection and pressing to produce the required details. It is planned to use such materials in the automotive industry. Although nonwoven fabrics have lower mechanical strength than woven fabrics, their production is the fastest and cheapest method of how to reuse short fiber [31].

A similar solution was presented by SGL Carbon GmbH from Wiesbaden (Germany). Waste carbon fiber is processed into nonwoven fabrics which are used for the roof of BMW i3, using the RTM method. It is also possible to use nonwoven fabrics in organic sheet production (with thermoplasts) or in SMC [33].

Shocker Composite company (USA) developed a method of recovering carbon fiber from a composite, from unhardened prepreg (duroplast matrix) using solvolysis in a continuous process. However, obtained fibers are fluffy and should best be used as pellets in thermoplastic matrices (ABS, PA).

A material with 20–40% fiber content that can be injected and used in large scale 3D printing was developed. Printing filaments were 1.75 and 2.85 mm in diameter. The inventors claim that their products have strength at the level of new fibers and their price is much lower. Industrial tests of 3D printing ended with success [34]. The company was set up by a doctoral student of Witchita State University (USA). The firm targets other customers than producers of brand-new fiber would. The company says that most big producers are reluctant to use recycled carbon fiber (rCF). The company wants to sell recovered fiber to small companies that manufacture high-end niche products using injection or pressing, e.g., biomedical products.

ABS, PA, and PP were used to inject mats 20 cm wide and 2 mm thick. The obtained fiber degree of order was up to 80%. Mats can be connected into wider elements whose width is limited only by the size of equipment [28]. Additionally, the company claims that their method of fiber recovery enables multiple uses of the material. This sets quite a new perspective for the whole industry [28].

R&M International developed a method of producing thermoplastic sheets reinforced with fiber reclaimed with Shocker Composite know-how. Sheets can be thermoformed or compressed into the final products [34].

Since 2015, the University of Kaiserslautern (Germany) has run the InTeKS project to use carbon fiber for carbon-reinforced organic sheets with good formability [28]. The problem is that continuous carbon fiber has an elongation at a break of 2% and flat organic sheets cannot be used to form 3D structures. Ultimately, fibers made of used bobbins, cut and separated from the roving will be woven into the yarn together with PA6 fiber. The resulting fabric can be 3D formed into organic sheets. Several European companies are involved in the project [28].

The same institute in collaboration with ELG Carbon Fiber and Honda R&D Europe developed a method of producing reinforcing tapes from pyrolysis recovered fiber for automated stacking into molds [35]. Composites were produced using RTM (Resin Transfer Moulding, saturation with a resin of dry reinforcing fibers in a closed mold) and VARI (Vacuum Assisted Resin Injection, kind of RTM processing method, vacuum helps resin to flow into the mold) technologies. Although fibers were not prepared, had a low length (compared to continuous fibers), and imperfect orientation, obtained composites had a strength of 38% and stiffness of 68% relative to original composites with long fibers. Efforts are underway to improve fiber orientation and to increase scale [35].

An interesting solution was suggested by Prodrive Composites. The company developed a new polymer matrix for carbon fiber composites. It is a reactive thermoplastic polymer that undergoes depolymerization during recycling. The material can be formed without an autoclave and fiber can be used many times. After the first forming process elements have the biggest strength and can be used for components working under heavy load. When the exploitation time of the first product is over, fiber and most of the matrix can be recovered with thermal or chemical degradation. Material obtained in this way can be used for further processing, e.g., for elements that do not work under heavy loads. When that material reaches the end of its life, it can be shredded and recycled. The third process can be repeated several times [36].

Table 5 presents a brief overview of the discussed recovery technologies with their advantages and disadvantages.

## 6. Recycling of Polymer Composites—EU Legislation

The leading international organizations involved in fiber production and recycling, such as The European Composites Industry Association (EuCIA) [37], The European Plastics Converters (EuPC) [38] and The European Composite Service Company (ECRC) [39], based on European Parliament and EU Council directives adopted and published in June 2011 a common standpoint saying that ”glass fiber reinforced thermosets are both material and energy recyclable through the cement kiln route and compliant with the EU legislation” [40].

The position was agreed upon, based on the End-of-Life Vehicles Directive 2000/53/EC [41] and the Waste Framework Directive 2008/98/EC [42].

End-of-Life Vehicles Directive (2000/53/EC) [41] specifies how to prevent waste from being generated from vehicles. It also sets out how to recycle, reuse, and recover parts from vehicles retired from use, how to limit waste to be neutralized, and improve activity aiming at environmental protection. The directive stipulates that from 1 January 2015 on a minimum of 95% of materials of end-of-life vehicles should be recovered. It also requires at least 85% of the average mass of a vehicle be available for reuse and recycling.

European Waste Framework Directive (2008/98/EC) [42] defines the basic concepts and definitions related to waste management. It emphasizes the need for increased recycling and highlights the reduced availability of landfills. It also establishes the waste hierarchy (prevention, re-use, repurpose, recycling, recovery, disposal).

On 14 June 2018, four directives changing the EU legislation on waste management were published in the Official Journal of the EU. The acts became effective on 4 July 2018 and all member states are obliged to pass national law accordingly. The directives set out new rules affecting:

- end-of-life vehicles, used electrical and electronic equipment, batteries and used batteries,
- waste,
- waste disposal,
- packaging waste.

According to the European Parliament and EU Council Directive of 30 May 2018 [43] changing directive 2008/98/EC on waste, starting from 1 January 2025 it will be required to selectively collect such waste categories as textile and hazardous materials. It will be mandatory to selectively collect bio-waste starting from 31 December 2023.

European Parliament and EU Council Directive of 30 May 2018, changed the earlier directive 1999/31/EC on waste disposal [44] and made it mandatory to reduce by 2035 the amount of municipal waste to 10% of its total volume and to put in place a working quality control system as well as waste disposal monitoring ability.

The biggest changes formulated in EP and Council directive 2018/852 of 30 May 2018, that changed directive 94/62/EC on packaging and packaging waste [45] said that it is mandatory to increase packaging waste recycling up to 65 wt% and 70 wt% by 31 December 2025 and 31 December 2030, respectively.

However, changes introduced in EP and Council directive 2018/849 of 30 May 2018 [46] altering legislation on end-of-life-vehicles (directive 2000/53/EC of 18 September, 2000), used electrical and electronic equipment (EP and Council directive 2012/19/EU of 4 July 2012), batteries and used batteries (EP and Council directive 2006/66/EC of 6 September 2006) primarily address the objectives of new rules and set out how to change national law to implement the directives and to implement the so-called circular economy.

There is no European legislation regarding the recycling of wind turbine blades. It is postulated that relevant acts should be formulated and passed. This would be helpful for composite material producers and users.

Global Wind Energy Council (GWEC) states that modern wind turbines are much larger than those built in the 1980s. The longer the blades, the higher demand for construction materials. Available sources indicate that the annual production of fiber composites exceeded 30 billion tons. If the average life span of a turbine blade is 20–25 years, after 2035 there will be approximately 225,000 t of used blades globally available to be recycled. There are additional composites from other applications. Therefore, after 2040, 380,000 t of glass composites will have to be utilized annually. Ultimately, carbon composites will also have to be recycled and their current annual production is 27,000 t.

A proposal for international guidelines for the dismantling and decommissioning of wind turbines is indispensable not only in the EU.

Recently, the European Chemical Industry Council (Cefic), the European Composites Industry Association (EuCIA), and WindEurope recommended the best strategies for the recycling of wind turbine blades [47], also the Federal Environment Agency formulated recommendations for the set-up of an efficient dismantling system in Germany [48].

Trends in legislation tend to increase producer responsibility, increase recycling rates, and reduce the availability of landfill. The European Union (EU) Circular Economy Package and the Waste Framework Directive limit waste to landfill but it is not clear yet how this will affect industrially derived and construction waste [49].

## Figures and Tables

**Table 1:** Comparison of experimental and theoretical values of modulus of composites with recycled particles [4].

Material No.	c_f_	c_r_	c_p_	Modulus Experimental (GPa)	Modulus Theoretical (GPa)
1	0.19	0.72	0.06	7.12	7.12
3	0.15	0.80	0.05	6.05	6.68
5	0.17	0.76	0.05	7.05	6.74
13	0.22	0.69	0.07	7.48	7.73
14	0.18	0.77	0.05	6.68	6.88

c_r_—volume fraction of resin.

**Table 2:** Production of polymer composites in Europe, divided by countries [7].

Country	2015 (kt)	2016 (kt)	2017 (kt)	2018 (kt)	2019 (kt)
Germany	212	220	226	229	225
Spain/Portugal	156	158	161	167	166
Italy	150	154	158	162	161
UK/Ireland	150	152	153	155	155
France	108	110	112	115	114
Belgium/Netherlands/Luxembourg	44	45	46	46	45
Finland/Norway/Sweden/Denmark	39	40	40	40	39
Austria/Switzerland	18	18	19	19	19
Eastern Europe *	192	199	203	208	217
Sum	1069	1096	1118	1141	1141

* Poland, Czech Republic, Hungary, Romania, Serbia, Croatia, Macedonia, Latvia, Lithuania, Slovakia, and Slovenia.

**Table 3:** World production of carbon fibers and composites [7].

Years	Production of Carbon Fibers (kt)	Production of Carbon Fiber Composites (kt)
2010	33.0	51
2015	58.0	91
2016	63.5	101
2017	70.5	114
2018	78.5	128
2020 *estimated*	98.0	160
2022 *estimated*	120.5	199

**Table 4:** The use of composites reinforced with carbon fibers by application in 2018 [7].

Business Line	Worldwide Demand		Worldwide Turnover	
	(kt)	(%)	bn USD	(%)
Aviation and Aerospace incl. Defense	55.31	36	12.91	56
Automotive	37.13	24	4.17	18
Wind energy	20.88	13	1.91	8
Sport and Leisure	20.11	13	2.55	11
Construction	7.74	5	0.46	2
Others	13.54	9	1.15	5

**Table 5:** Summary of carbin fiber (CF) recovery technologies.

Recycling of Dry Fibers		
Technology	Advantages	Disadvantages
Non-woven textile recycling/mechanical recycling	- fastest and cheapest method of how to reuse short fibers - non-woven fabrics can be used in organic sheet production (with thermoplasts) or in SMC - different sizes of recyclates can be recovered - non-woven material can be used in different industries—e.g., the automotive industry to improve the resin flow during injection	- non-woven fabrics have lower mechanical strength than woven fabrics
**Chemical process**		
Solvolysis e.g., Shocker Composite company	- from unhardened prepreg (duroplast matrix) - continuous process which makes upscaling possible - used in large scales for 3D printing - possible solution for approved cradle-to-cradle approach with composite material	- obtained fibers are fluffy and should best be used as pellets in thermoplastic matrices - numerous lab-scale experiments have been carried out, but only a few studies have reached industrial or semi-industrial scale
**Thermal process**		
Pyrolysis method e.g., ELG Carbon Fiber and Honda R&D	- allows the recovery of fibers, fillers and inserts - can process up to 5 tons a day - ground fiber suitable for molding compounds and coating (80–100 µm) - cut fiber good for thermoplast reinforcement (3–100 mm) - fiber suitable for products with the basis weight of 100–500 g/m2 (60–90 mm)	- products are not meant to replace new carbon fibers - especially glass fibers suffer from high temperature and their mechanical properties can decrease by at least - 50%
